# Square-slotted THz metamaterial-inspired MIMO antenna design optimized with machine learning for TWPAN networks and next-generation communication systems

**DOI:** 10.1038/s41598-026-41207-w

**Published:** 2026-03-03

**Authors:** Meshari Alsharari, Yogesh Sharma, Ammar Armghan, Khaled Aliqab, S. K. Patel, Aymen Flah

**Affiliations:** 1 Department of Electrical Engineering, College of Engineering, Jouf University, 72388 Sakaka, Saudi Arabia; 2 Department of Physics & Environmental Sciences, Sharda School of Engineering & Science, Sharda University, Greater Noida, 201310 Uttar Pradesh India; 3 Department of Computer Engineering-AI & DB, Marwadi University, Rajkot, 360003 Gujarat India; 4 Applied Science Research Center, Applied Science Private University, Amman, 11931 Jordan; 5 ENET Centre, CEET, VSB-Technical University of Ostrava, Ostrava, Czech Republic; 6 National school of engineering of gabes, University of Gabes, Gabes, 6072 Tunisia

**Keywords:** THz, Antenna, MIMO, Metamaterial, Diversity parameters, Ultra-broadband, High gain, THz Wireless Personal Area Network (TWPAN), Engineering, Materials science, Physics

## Abstract

The growing demand for high-speed communication necessitates antennas operating at higher frequencies in the THz range with broad bandwidth, compact size and higher gain. In this research, we have proposed a compact THz antenna designed with metamaterial-inspired elements to achieve improved gain and wideband performance. This paper presents the design and performance analysis of a high-efficiency MIMO antenna system for terahertz (THz) communication applications. The proposed antenna is optimized to deliver robust diversity performance, low mutual coupling, and minimal channel degradation. The design is also optimized with machine learning, with the highest R^2^ value of 0.95. The optimized design gives 54 THz bandwidth and 7.6 dBi gain. The ECC remains well below 0.005, ensuring excellent isolation between elements. The TARC exhibits values below − 10 dB across the critical bandwidth, confirming low reflection under simultaneous port excitation. These results demonstrate the designed structure’s potential for integration into THz communication and THz Wireless Personal Area Network.

## Introduction

Antenna design has gone a long way from bulkier horn antennas to tiny patch antennas. The research on antennas is evolving day by day as we are moving towards THz communications. THz communication antennas need ultrabroad bandwidth and high gain to be operational at high-speed THz networks. The demand for ultra-fast, low-latency, and high-capacity communication systems has led to significant research into the design of antennas operating in the terahertz (THz) band for THz applications^1^. THz antennas are a crucial enabler of the envisioned THz networks due to their ability to support extremely high data rates and dense device connectivity. However, designing efficient THz antennas needs precise dimensional requirements which can be met with lithography techniques^2^. Ikram et al. present a comprehensive review that bridges current 5G antenna technologies and the anticipated requirements for THz communication systems. The paper critically evaluates state-of-the-art advancements in antenna designs, focusing on compactness, reconfigurability, and performance at millimeter-wave and sub-terahertz frequencies. The review highlights the pivotal role of Massive MIMO, beamforming, metasurface-based antennas, and planar phased arrays in enabling high-data-rate communication^3^. Rasilainen et al. provide an in-depth review of the critical hardware challenges and design considerations for sub-terahertz designs, which are enablers for future THz communication systems. The study investigates the practical limitations and trade-offs in designing high-gain, wideband, and energy-efficient antenna systems that operate effectively in the 100–300 GHz frequency range^4^. Researchers proposed an O-shaped fractal antenna optimized specifically for THz mobile communication devices. The antenna’s geometry is derived from recursive fractal iterations that allow for efficient space utilization, resulting in miniaturized structures without compromising performance. The design emphasizes two key requirements for next-generation devices: broad bandwidth and high gain, which are critical for supporting dense data transmission in THz and sub-THz frequency bands^5–7^.

In their comprehensive review, Hussain et al. present an in-depth exploration of metamaterials (MTMs) and their vital position in improving the performance of reconfigurable antennas (RAs). The study emphasizes the ability of MTMs to overcome traditional antenna design limitations, particularly in terms of bandwidth, gain, size, and radiation pattern agility, making them ideal for next-generation wireless systems, including emerging THz applications. The integration of these MTMs into antenna designs leads to substantial improvements in miniaturization, frequency tunability, and radiation control^8^. Hui et al. design a metamaterial-inspired superstrate using V-shaped resonator elements arranged symmetrically in a double-V configuration. This structure exhibits polarization conversion characteristics, effectively altering the incident wave’s polarization to match the receiving antenna’s polarization. As a result, the proposed design enables dynamic polarization tunability, allowing for improved energy absorption from various incident wave directions and polarization states^9^.

MIMO metamaterial antennas can be used for the improvement of gain, bandwidth and size^10^. Khan et al. propose a dual-band MIMO antenna design tailored for 5G applications, with a focus on mutual coupling reduction through the integration of metamaterials. Mutual coupling between closely spaced antenna elements is a major challenge in compact MIMO systems, as it negatively impacts diversity performance and overall system capacity. The study addresses this issue by incorporating a metamaterial-based decoupling structure, leading to improved isolation and radiation characteristics^11^. Hasan et al. introduce a novel compact wideband MIMO antenna design integrated with Mu-Near-Zero (MNZ) metamaterials aimed at enhancing performance for 5G New Radio applications. The research addresses key design challenges in modern wireless systems, such as achieving high gain, broad bandwidth, low mutual coupling, and compact size, which are vital for efficient 5G deployment. The core innovation of the design lies in the use of MNZ metamaterial structures, which possess near-zero permeability (µ ≈ 0) at specific frequencies. These materials enable enhanced electromagnetic field manipulation, leading to significant improvement in gain and radiation efficiency, while simultaneously reducing mutual coupling between closely spaced MIMO elements. The integration of MNZ materials helps in suppressing surface wave propagation and redistributing the energy constructively in the far-field region^12^. A tree-shaped micro-scaled MIMO antenna with the polyimide substrate size of 600 × 300 µm2, which has been operated in the impedance bandwidth of 0.276–0.711 THz. The isolation of -52 dB has been noted between the radiating element of MIMO antenna^13^. The wideband of 0.35 to 0.75 THz MIMO antenna, which is based on graphene characteristics have been determined with the results of -25 dB isolation^14^.

Machine learning algorithms can be used with metamaterial MIMO antenna designs to improve different antenna parameters. Machine learning optimization can be applied to improve the antenna parameters. Pandey et al. present a cutting-edge study on MIMO structure dielectric resonator antennas, targeting the 5G communication. The novelty of this work lies in the optimization using Machine learning, thereby reducing the iterative and computationally expensive nature of conventional electromagnetic simulations^15^. Li et al. present a significant advancement in antenna optimization by introducing XGBoost algorithm. Traditional antenna design and optimization processes often suffer from high computational costs due to iterative full-wave electromagnetic simulations. To address this limitation, the authors develop a novel surrogate-assisted machine learning framework that operates in an online learning environment, enabling continuous performance improvement during the design process^16^. M. A. Haque et al. propose an innovative design and performance enhancement strategy for millimeter-wave 5G MIMO antenna arrays by integrating machine learning techniques for gain prediction. The study addresses critical challenges in 5G antenna design, including high data throughput, wide bandwidth, and compact form factor, while ensuring high gain and low mutual coupling among multiple antenna elements^17^.

The proposed square slotted THz metamaterial-inspired MIMO antenna has the following novelties:

I. Metamaterial-Inspired Geometry: The square‑slotted design introduces engineered resonances that recapitulate metamaterial behaviour, which enables enhanced confinement and broadside radiation at THz frequencies without requiring strict homogenization.
II. Machine Learning-based optimization: In contrast to the traditional parametric sweeps, or gradient evaluation, when a machine learning is incorporated, the multidimensional design space is optimally explored to achieve optimal impedance matching, isolation, and gain simultaneously.
III. High Performance MIMO Configuration: The MIMO configuration is a two port one that provides low mutual coupling and high isolation, which is demonstrated by the S parameter analysis and therefore suitable in any reliable short range THz communication.
IV. Application to TWPAN and Beyond: The antenna is precisely designed to serve the purpose of Terahertz Wireless Personal Area Networks (TWPAN), which is the next generation in communication, including high data rates, compactness and directionality.
V. Solver Independent Robustness: The machine learning system has the ability to generalize design trends in the parameter space, eliminating reliance on a single simulation tool and guaranteeing solver independent reliability.

The works discussed so far display that the necessity for high-speed communication requires an antenna with high gain and a broad bandwidth. We have proposed a metamaterial-inspired antenna that is optimized with machine learning to be applicable for smart and high-speed devices. The following sections give the design and results overview.

## Antenna design

**Fig. 1:**
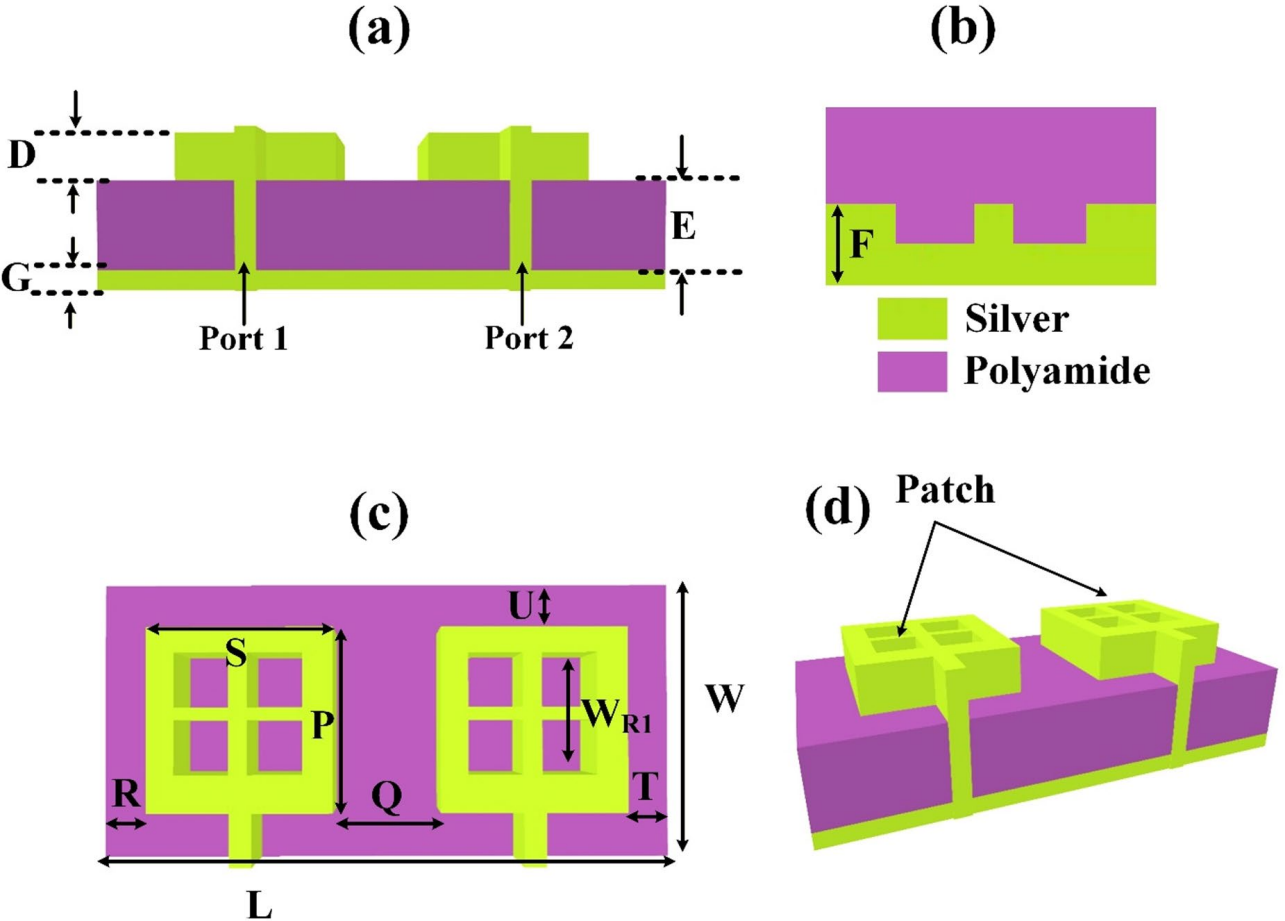
(**a**–**d**) Two-port MIMO Design with different views. Silver patch, Polyamide substrate. The length of the patch is kept low at 35 × 35 µm^2^.

The metamaterial MIMO antenna design created by applying the four different square slots in a square patch 2-port MIMO antenna is investigated and presented in Fig. 1. The design shows the different views of the design. Recent advances in high-frequency antenna and metamaterial-inspired design have increasingly utilized silver patches due to their excellent electrical conductivity and stable surface characteristics. The integration of a ground plane plays a crucial role in minimizing back radiation and enhancing directional performance. Among various substrates, polyamide has gained attention due to its flexibility, making it suitable for conformal and high-frequency applications. For instance, researchers have reported the development of flexible antennas and metasurfaces on polyamide films that maintain performance under mechanical deformation, especially in 5G and THz frequency bands. The synergy between silver conductive layers and polyamide substrates enables low-profile, high-gain, and broadband antenna systems suitable for wearable electronics, THz sensing, and future wireless communication systems. Moreover, the etched ground plane approach allows for tailored current distributions, leading to enhanced bandwidth and gain performance. The design is prepared with compact dimensions of a 35 × 35 µm^2^ single patch element. The height of the structure is also kept smaller to 0.5 μm, 1.6 μm and 1 μm for the three-layer structure. The overall substrate size is 110 × 55 µm^2^. The ground plane is defected by keeping the width of the ground to only 25 μm. The edge-to-edge distance between two radiating elements is 20 μm, and diatance from the patch to the substrate end is 10 μm. The dimensions are not up to the scale. All the physical parameters are optimized and optimization is provided in the results analysis section. Different parameter values of the structure are given in Table 1.

**Table 1:** Design parameters.

Parameter	P	S	W_R1_	D	E	F	G	L	W	Q	R	T	U
Size (µm)	35	35	25	0.5	1.6	25	1	110	55	20	10	10	10

**Fig. 2:**
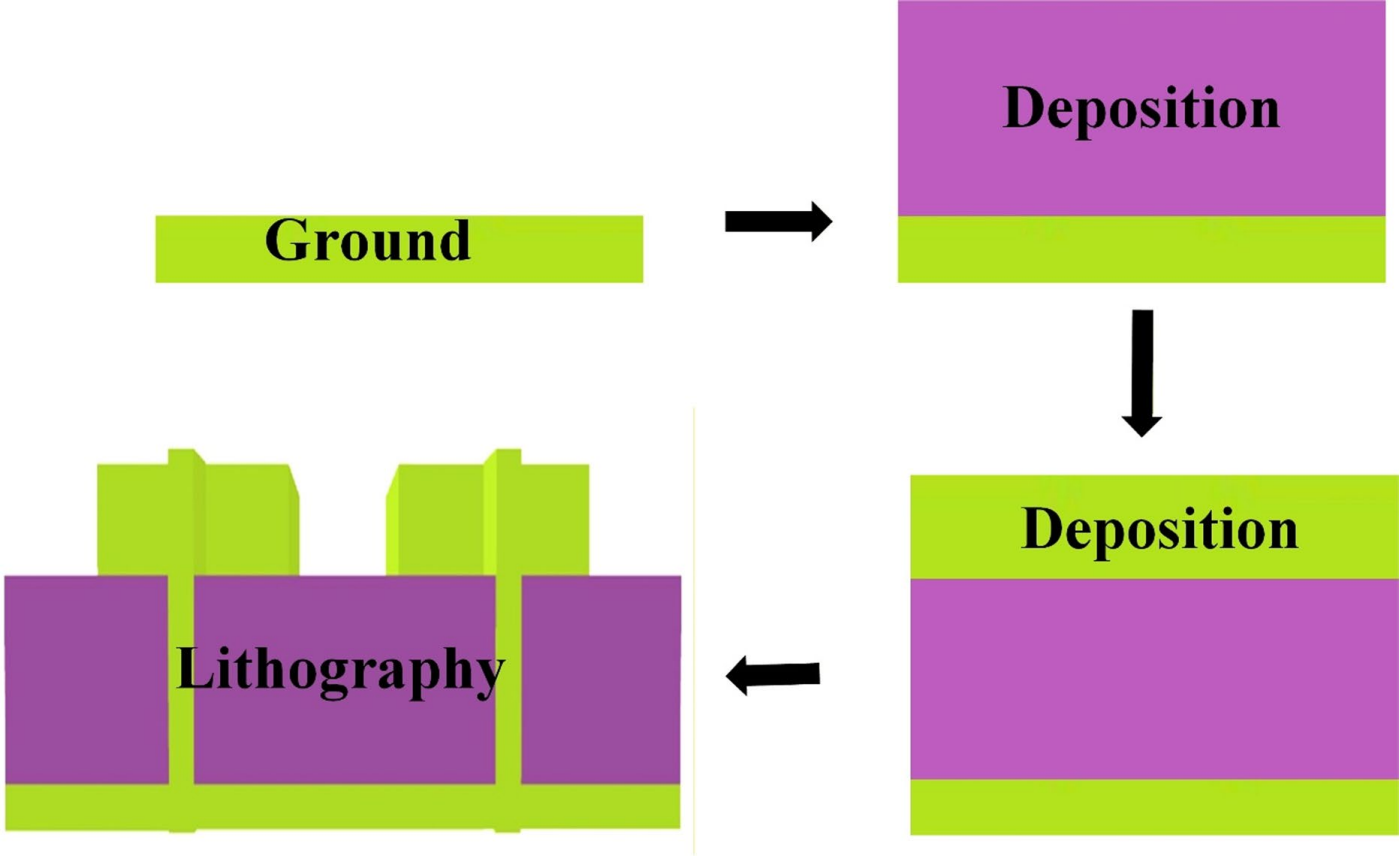
Fabrication approach. Ground plane for reflection control, Polyamide dielectric for flexibility and low loss, Silver or other metals for high-conductivity structures and lithography for precise patterning.

Figure 2 illustrates the fabrication process of a multilayer antenna or metasurface structure, involving a ground plane, dielectric substrate, and silver patch, using deposition and lithography techniques. A base layer is prepared, likely made of a conductive material (e.g., silver or copper), which acts as the ground plane. A dielectric substrate layer, such as polyamide, is deposited on top of the ground plane. This layer acts as the insulating medium between the ground and the top conductive pattern. This serves as the bottom conductive surface of the antenna or device. Another layer is deposited, possibly for: Multilayer structuring. This could also be for encapsulation or to form a MIM (metal–insulator–metal) structure. Photolithography is used to define specific metallic patterns (e.g., slots, patches). A photoresist is applied, exposed, and developed to etch or pattern the metal layers as per the design. This step finalizes the antenna or metamaterial geometry, creating slots or resonators on the top layer. This process is typical in MEMS, RF, or antenna fabrication, especially for flexible or high-frequency devices like THz antennas, THz sensors, or SPR biosensors.

The resonance presented by the slot geometry controls the functioning of the square slotted THz metamaterial MIMO antenna and presents multiple current routes, as well as allows operating modes to mode change in the target band. The energy is excited on these slots via the feed line and generates high local values that radiate more or less in the broadside direction. The close distance between and the orientation of the elements during the MIMO arrangement lowers the mutual coupling with the same impedance validated by the S parameter analysis. The optimization process of machine learning is essential in the refinement of slot sizes and feed parameters and thus a compromise between gain, isolation and stability of the impedance. This is a mechanism, which provides high gain and constant directivity in the antenna, and it is deemed suitable in short-range communication applications at THz frequencies.

Although this suggested square-slotted THz metamaterial-inspired MIMO antenna has demonstrated impressive simulated efficiency, it is significant to recognize limitations as well. The current research study focuses mainly on computational simulation along with machine learning to improve efficiency, and does not include practical fabrication or measurements at THz frequency ranges. The practical implications of these kinds of frameworks are frequently facing challenges due to manufacturing tolerances, structural defects, and orientation precision, all of which might impact resonance performance and isolation features. Some of these variables may result in differences between simulated and actual outputs, specifically in the THz frequency range, where even small geometrical imperfections might have severe effects on functionality.

### Metamaterial analysis

Analysis is presented in Eqs. (1–5)^18,19^. Equation (1) is used to extract the normalized impedance (Z) of a material from its measured or simulated reflection (S₁₁) and transmission (S₂₁) coefficients. The expression is derived from the transmission line model of the material under test and relates the electromagnetic boundary conditions to the S-parameters. The ambiguity in sign (±) is typically resolved by enforcing physical constraints like positive real impedance. Equation (2) represents the exponential transmission phase term across a slab of thickness dd, where *n* is the complex refractive index, and *k*_*0*_​ is the free-space wave number. This formulation accounts for multiple internal reflections and matches the boundary conditions of the S-parameter model. It’s used to calculate the complex phase factor from which the refractive index is derived. Equation (3) is used to extract the complex refractive index *(n*) of the metamaterial. The real part of *n* is retrieved by taking the imaginary part of the logarithm of the phase factor, corrected by an integer mm to resolve the branch ambiguity in the logarithm. The imaginary part (which indicates losses) comes from the real part of the logarithm. Equations (4, 5) gives the value of permittivity (ε) and permeability (µ) from n and Z.

1 $$\:Z=\pm\:\sqrt{\frac{{\left(1+{s}_{11}\right)}^{2}-{s}_{21}^{2}}{{\left(1-{s}_{11}\right)}^{2}-{s}_{21}^{2}}}$$

2 $$\:\:{e}^{in{k}_{0}d}=\frac{{s}_{11}}{1-{s}_{11}\frac{2-1}{2+1}}$$

3 $$\:n=\frac{1}{{k}_{0}d}\left\{\right[ln{e}^{in{k}_{0}d}\left)\right]{\prime\:}{\prime\:}+2m\pi\:\}-i[ln\left({e}^{in{k}_{0}d}\right)\left]{\prime\:}\right]$$

4 $$\:\epsilon\:=\frac{n}{z}$$

5 $$\:\mu\:=nz$$

## Result analysis

**Fig. 3:**
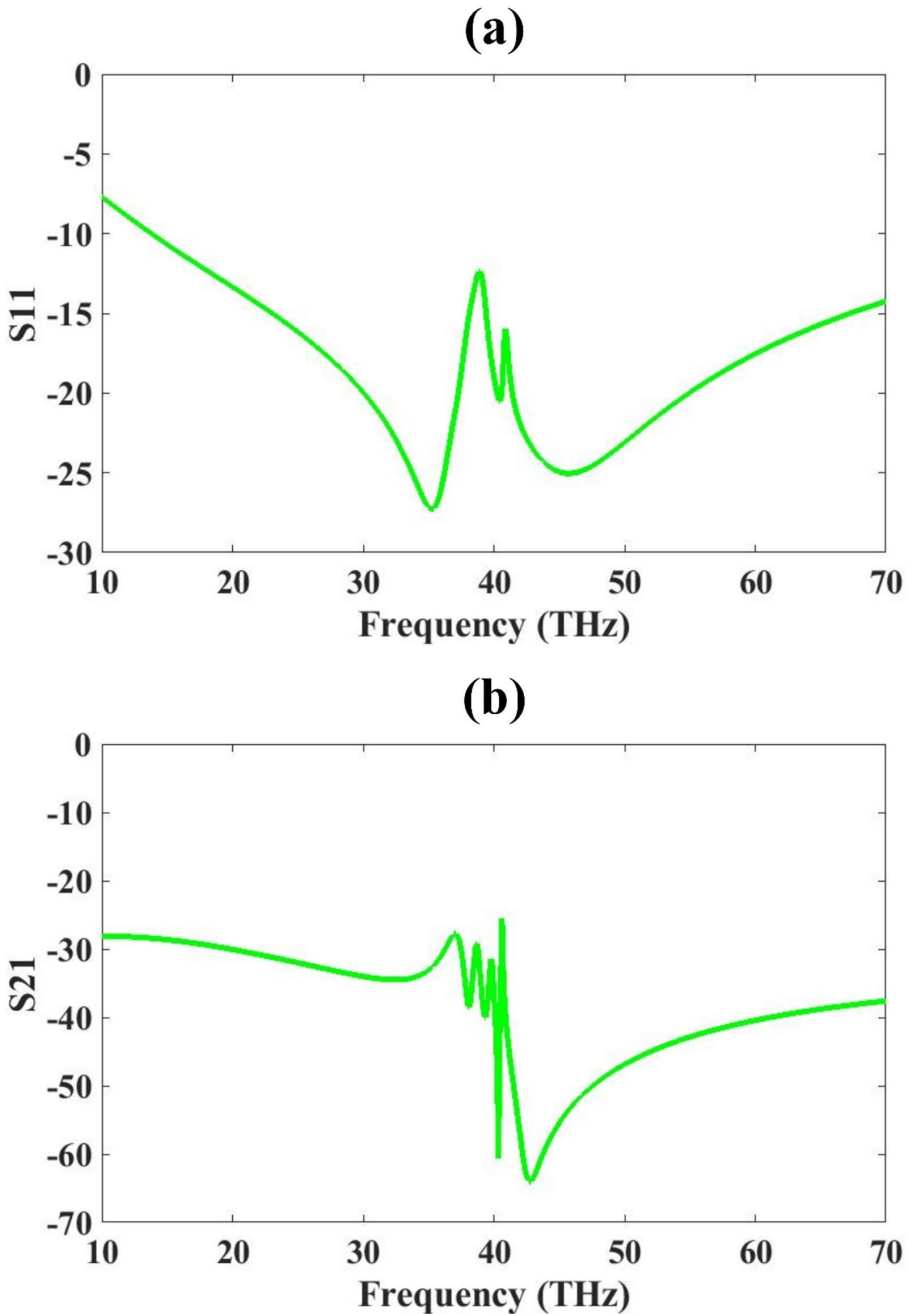
S-parameters of the two-port MIMO design (**a**) S11 (**b**) S21.

**Fig. 4:**
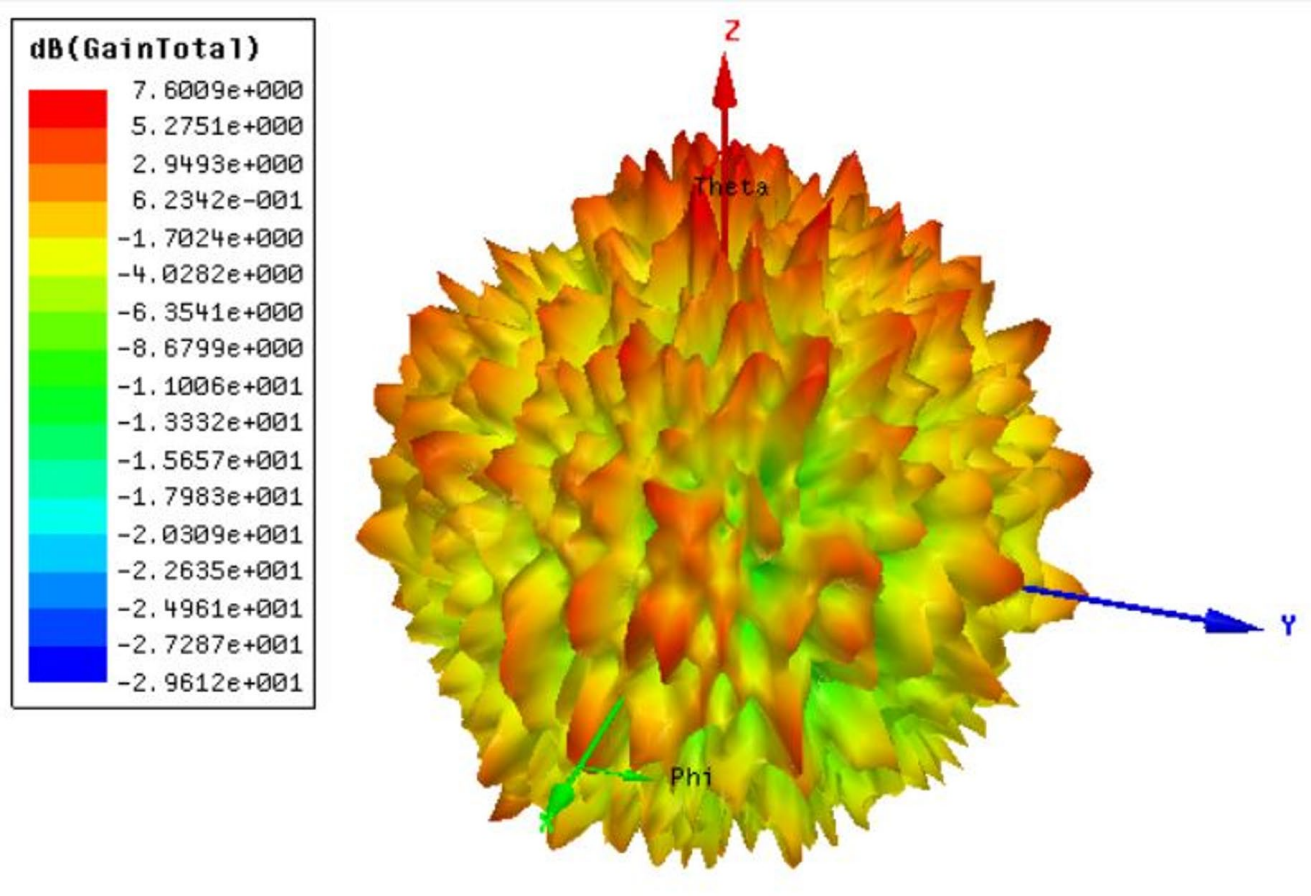
Gain polar plot of the two-port MIMO design. 7.6 dBi highest gain available.

**Fig. 5:**
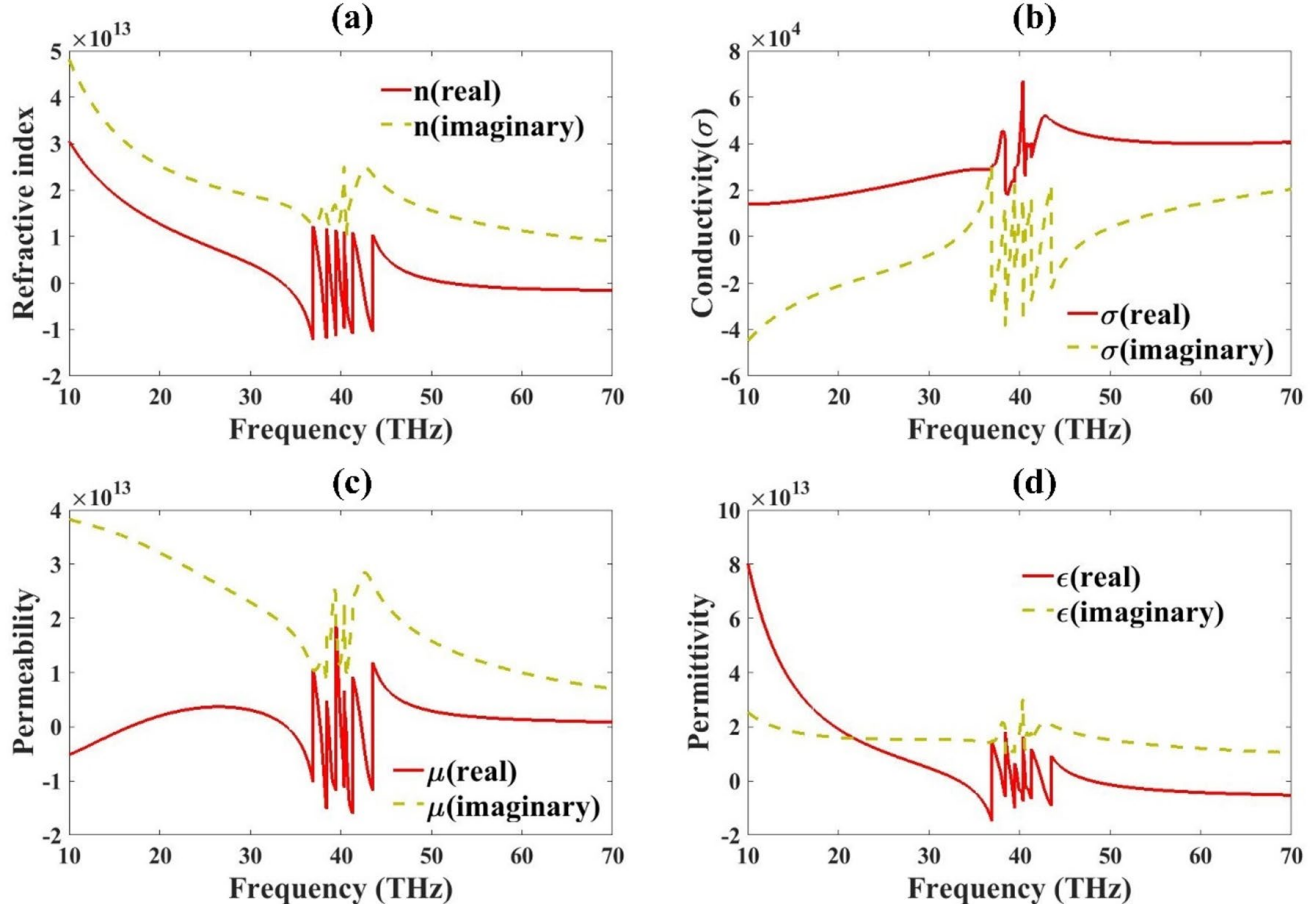
Metamaterial antenna properties (**a**) n, (**b**) σ, (**c**) µ and (**d**) ε.

**Fig. 6:**
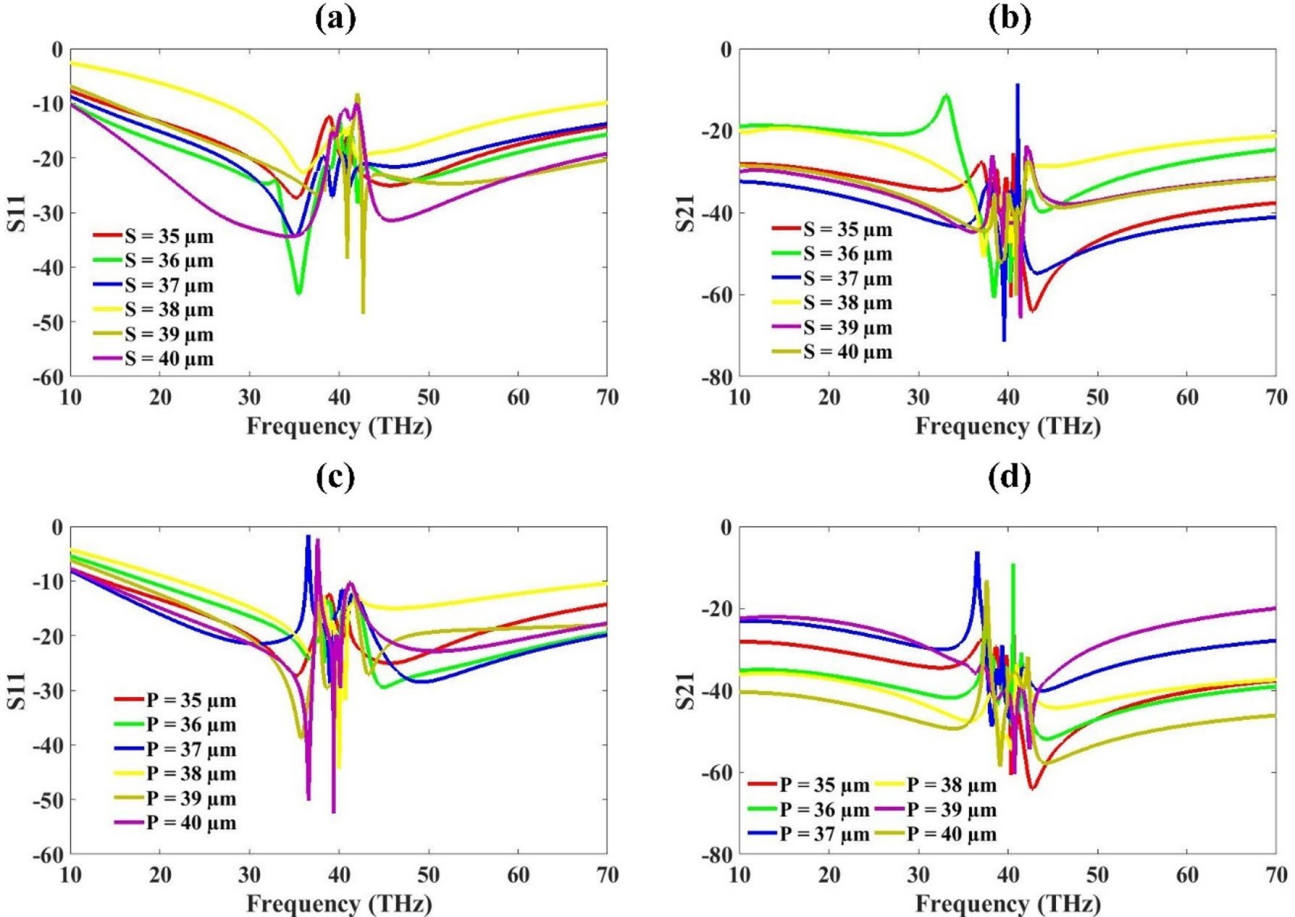
S-parameter for variation in length (S) (**a**) S11 (**b**) S21, Width (P) (**c**) S11 (**d**) S21.

**Fig. 7:**
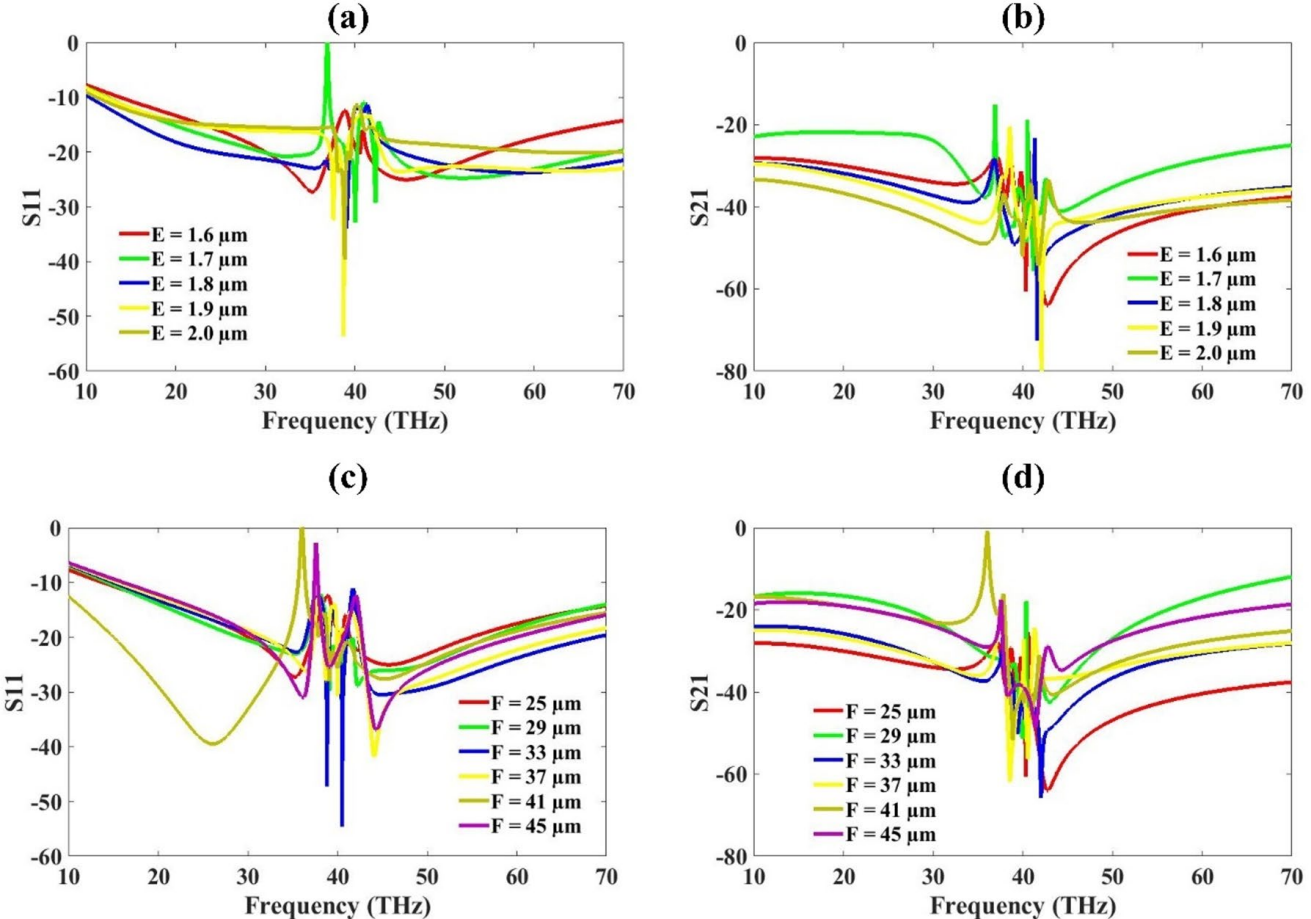
S-parameter for variation in substrate height (E) (**a**) S11 (**b**) S21, Ground Plane Width (F) (**c**) S11 (**d**) S21.

**Fig. 8:**
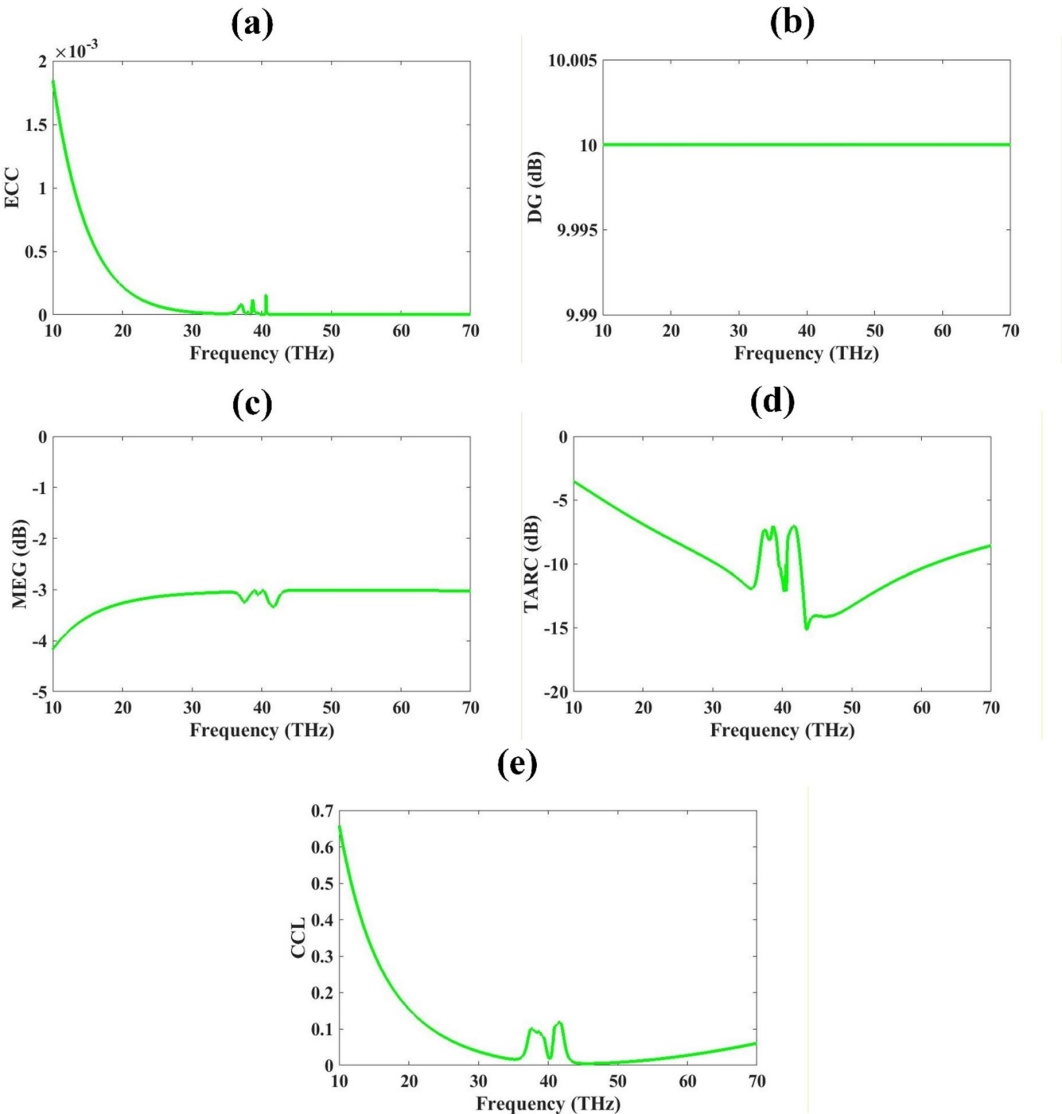
(**a**–**e**) MIMO performance Parameters. Lower ECC, Higher DG gives good isolation. MEG is less than negative 3dB, and TARC is less than negative 5dB for most of the range. CCL is 0.1 bits/Hz.

**Fig. 9:**
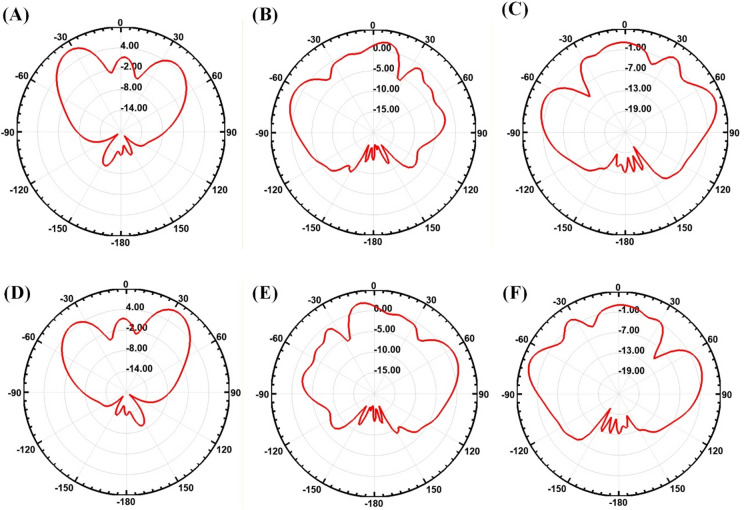
(**A**–**F**) Radiation pattern of the proposed square-slotted metamaterial MIMO antenna.

**Fig. 10:**
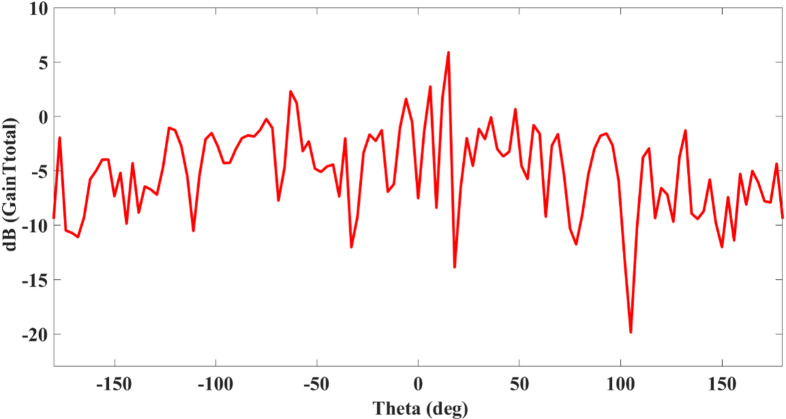
Realized gain plot of the proposed square-slotted metamaterial MIMO antenna.

**Fig. 11:**
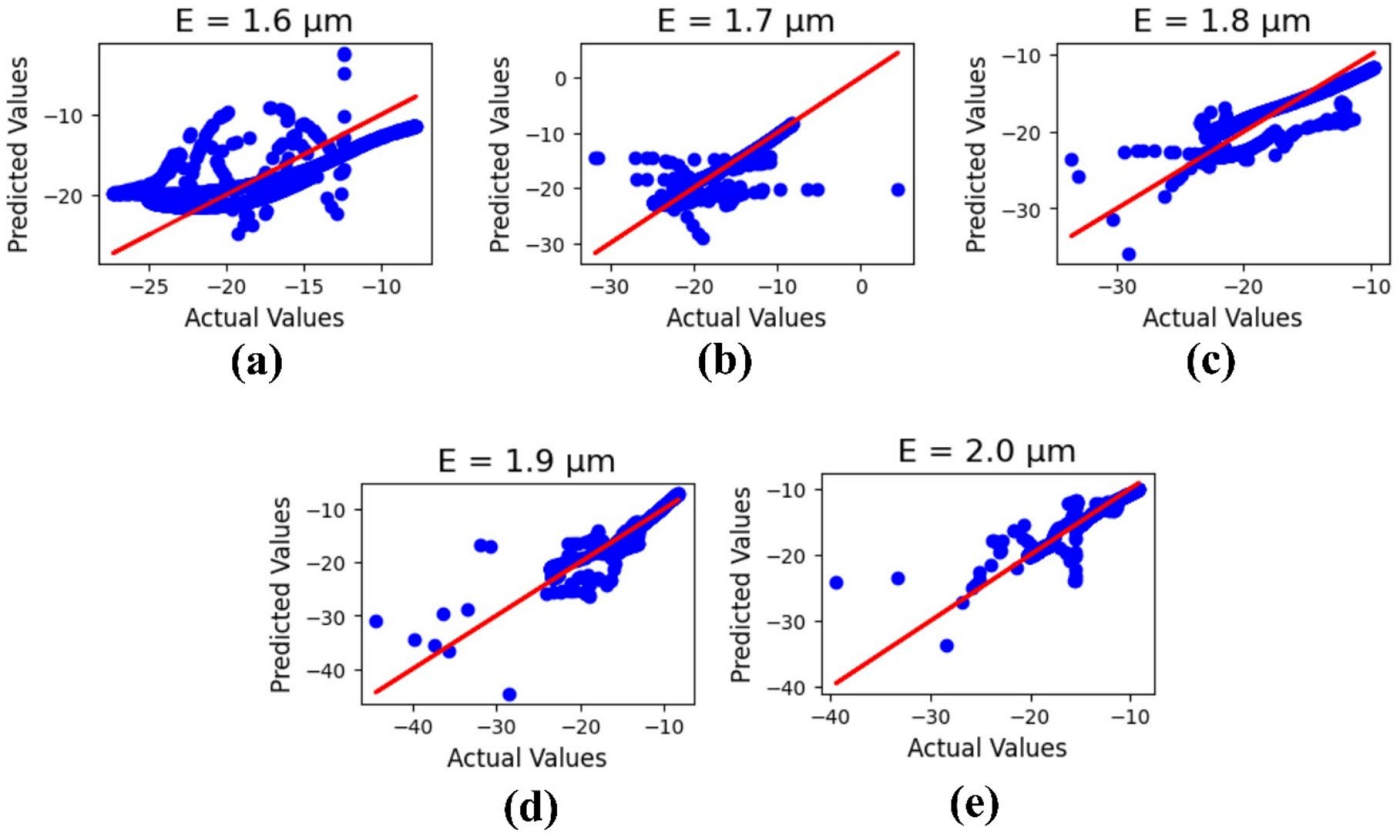
ML-based optimization results for substrate height variations, shown for parametric values in micrometers: (**a**) 1.6, (**b**) 1.7, (**c**) 1.8, (**d**) 1.9, and (**e**) 2.0.

The results are analysed and optimized using COMSOL Multiphysics and presented in Figs. 3, 4, 5, 6 and 7. The machine learning optimization and its results are presented in Figs. 8, 9, 10 and 11. The S-parameters are analyzed and presented in Fig. 3. The results show that S11 is showing a good response below − 10 dB for most of the investigated range from 14 THz to 70 THz, which gives a 56 THz bandwidth. S11 shows a minimum of − 27 dB at 35THz, indicating strong resonance and good matching. The S21 is also less than − 30 dB for most of the range and shows the lowest minimum values of -65 dB. S21 value is − 65 dB, which indicates extremely low transmission, meaning that very little or nearly no signal is being transmitted from port 1 to port 2. This indicates there is strong isolation between the two ports, which is essential for the MIMO antennas.

The gain polar plot results are analyzed in Fig. 4, which shows that the highest gain achieved for the design is 7.6 dBi.

### Metamaterial properties analysis

This section presents the electromagnetic parameters (σ, n, µ and ε) of a metamaterial-loaded antenna design, usually extracted using simulated scattering parameters (S-parameters) (Fig. 5). The first part gives the response for refractive index. The real part indicates how the phase of the wave propagates through the material. Negative real parts indicate left-handed behavior, common in metamaterials. The imaginary part indicates loss-ideally, it should be low to ensure efficient transmission. The sharp variations at certain frequencies imply dispersive behavior that may support resonance or filtering. Conductivity shows a good value for the whole range in the real part. The real part of permeability indicates whether the material supports magnetic response. Negative values indicate magnetic metamaterial behavior (µ-negative). The imaginary part shows magnetic losses. Spikes and negative regions near resonant frequencies suggest strong magnetic resonances, important for shaping the antenna’s radiation characteristics. The real part of the permittivity represents the electric polarizability. Negative values indicate electric metamaterial behavior (ε-negative). Again, peaks or transitions correspond to resonance. The imaginary part represents dielectric losses.

Figure 6 represents the S-parameters for the variation in length (S) and width (P) of patch. This variation in a structural parameter S and P (ranging from 35 μm to 40 μm). It is part of a parametric optimization study, which helps in selecting the optimal value of ‘S’ and ‘P’to achieve the best performance. The S₁₁ indicates how much power is reflected back from the antenna. Values below − 10 dB indicate good impedance matching. The region between 30 and 50 THz shows multiple dips across curves, which indicates multiple resonance frequencies for different ‘S’ values. These dips represent the operational bands of the MIMO antenna. For all the length values, the band is below − 10 dB but for 38 μm it is not showing a good response so we can take values other than that for the optimized values. The S21 parameter show that aprt from 40 μm to 36 μm, most of the values have S21 less than − 30 dB, which gives good isolation required for MIMO antenna. We have selected the values of 35 μm as it gives a good response for the length ‘S’ value, as well as its lower value gives compact size and lower cost.

The other parameter, which is investigated in this figure, is width (P). It is also investigated for the same variation range as that of length. Here also the 38 μm yellow line results show the lower value for S11 and blue line 37 μm for S21. Thus, apart from these two values, we can take any other. We have selected the optimized values of 35 μm because of compactness as well as low cost due to its lower value.

The plot shows the reflection coefficient (S11) in dB versus frequency (THz) for different values of substrate height E, ranging from 1.6 μm to 2.0 μm in Fig. 7. The S21 is also varied for the same range for a similar spectrum and results are presented in the figure. As E increases from 1.6 μm to 2.0 μm, the resonance frequency slightly shifts. Lower values of E (e.g., 1.6 μm, red line) show resonance peaks slightly shifted to higher frequencies. Higher values (e.g., 2.0 μm, olive line) show resonance at slightly lower frequencies. Substrate height (E) influences the effective dielectric constant and impedance of the antenna. Increasing E typically increases the stored electric energy, altering the resonance behaviour. Too thin or too thick a substrate can result in impedance mismatch, leading to higher reflections (worse S11). The optimal substrate thickness for the best S11 (minimal reflection) is around 1.9 μm. Increasing E improves bandwidth but may shift the resonance to lower frequencies. Designers should balance between low reflection, bandwidth, and resonant frequency shift when choosing substrate height.

The plot also shows the variation in ground plane width F values from 25 μm to 45 μm and its effect on the S-parameters. The red color plot in S11 and S21 is showing less than − 10 dB and − 30 dB for most of the investigated wavelengths. This plot is for 25 μm ground plane width. The other values also show similar response except 41 μm where the response is poor compared to to other values. The 25 μm is selected as the optimized value.

### Sensitivity to design parameter variations

The operational efficiency of the presented square-slotted THz metamaterial-based MIMO antenna has a severe sensitive to slight changes in essential structural parameters such as length and width of the antenna patch, substrate height, ground plane width, etc. A small modification in these variables might result in significant changes in the resonant frequency, impedance matching, and isolation values. For instance, a minor shift in patch length influences the efficiency of the present route, consequently resulting in the bandwidth and gain parameters. In a similar way, deviations from substrate height or its dielectric properties could impact coupling between adjacent components, subsequently affecting mutual isolation. This level of sensitivity becomes particularly significant at THz frequency ranges, when even minute mistakes in manufacture might trigger noticeable differences between simulated and real-life outcomes. Recognizing this, a machine learning-driven regression approach has been implemented to determine parameter ranges that retain constant efficiency, thus improving the structure’s adaptability to predictable fabrication and material fluctuations.

### MIMO performance parameters

Figure 8 illustrates the key MIMO performance parameters of the proposed antenna system in the terahertz frequency range (10–70 THz), including ECC, DG, TARC, MEG and CCL (a) ECC: The ECC values remain significantly below the acceptable threshold of 0.5, indicating excellent isolation and minimal mutual coupling between the antenna elements. This confirms superior spatial diversity characteristics. (b) DG: The diversity gain remains constant at approximately 10 dB across the operational bandwidth, demonstrating the antenna’s ability to maintain robust diversity performance and effectively mitigate multipath fading. (c) MEG: The mean effective gain fluctuates slightly around − 3 dB, which is within the acceptable range for MIMO systems, signifying balanced power reception across antenna elements in a multipath environment. (d) TARC: The TARC remains below − 10 dB across the majority of the frequency band, particularly between 35 and 45 THz, confirming low reflection and efficient operation when all ports are simultaneously excited, which is critical for practical MIMO deployment. (e) CCL: The channel capacity loss remains well below the critical value of 0.4 bits/s/Hz, with a minimum value under 0.1 bits/s/Hz observed in the operational band. This ensures that the system experiences minimal degradation in channel capacity due to correlation effects.

Figure 9 illustrates the radiation pattern of the proposed square-slotted metamaterial MIMO antenna. Figure 9A illustrates the radiation pattern at θ = 0°, Fig. 9B illustrates the radiation pattern at θ = 60°, and Fig. 9C illustrates the radiation pattern at θ = 120°. Furthermore, Fig. 9D illustrates the radiation pattern at θ = 180°, Fig. 9E illustrates the radiation pattern at θ = 240°, and Fig. 9F illustrates the radiation pattern at θ = 300°.

Figure 10 illustrates the realized gain plot of the proposed square-slotted metamaterial MIMO antenna. The realized gain has been plotted in the theta range of -180 (deg) to 180 (deg). The highest gain value of nearly 6 dB has been noted at the frequency value of 32 THz along with θ = 90°.

Comparison of our design with other designs is also given in Table 2, which shows that our design has good bandwidth and gain for the designed MIMO design with the lowest possible dimensions. This makes the design smaller and cost-effective.

**Table 2:** Comparison of different antenna parameters of our design with other references.

	Size (µm^2^)	Edge-to-edge distance of two radiating patches (µm)	Bandwidth (THz)	Gain (dBi)
^20^	110 × 130	55	0.78	7.5
^21^	1000 × 1400	-	9.6	19
^22^	2000 × 1000	-	76	10.43
^23^	-	-	0.13	8
^24^	13 × 26	-	18.18	1,5
^26^	8 × 32 × 0.25 mm^3^	-	24.0, 38.0, 44.43, 49.60, and 57.0 GHz	4.23
^26^	125 × 125	-	9.3	-
^27^	800 × 600	-	5.71	7.934
^28^	130 × 85	-	0.6	7.23
^29^	822 × 280	-	0.116	13.6
^14^	600 × 300	-	0.4	5.49
^30^	800 × 600	-	9	9.5
^31^	500 × 500	-	-	8.36
^32^	227.24 × 95.52	-	4.33	13.3
^33^	360 × 220	-	0.6	11.8
^34^	103 × 80	-	1.77	14.44
^35^	800 × 1170	-	14.8	-
^36^	90 × 30	-	6	12.38
^37^	130 μm × 240 μm × 12.5 μm	-	0.93	4
Four-square slotted MIMO antenna	110 × 55		66	7.6

## Machine learning-driven optimization

The algorithm for optimization technique implemented in the present research has its basis on a model of linear regression, which has been chosen for its ease of use, versatility, and feasibility to determine direct correlations between antenna parameters for design as well as efficiency metrics. In this sort of method, the geometrical parameters associated with the square-slotted metamaterial-based antenna have been investigated as independent predictors, with significant functional measures, which include gain, isolation, as well as bandwidth operating as dependent outputs. The values of the regression coefficient have been generated using a set of training data produced with computational simulations, which enabled the regression algorithm to detect linear patterns, along with offering accurate predictions about design variants. Although linear regression may not take into consideration more complex nonlinear relationships as much as the latest machine learning approaches, it serves as an easily understood and computationally effective way of improvement. The claims regarding performance improvement are therefore consistent with the adopted methodology, as the regression model effectively guided the identification of parameter ranges that yield enhanced antenna characteristics within the THz regime.

For the current antenna design, machine learning has been applied using linear regression to optimize performance^38^. In this analysis, the ML model evaluates the output efficiencies of different layer heights by comparing actual and predicted S11 values. The results demonstrate high R² values along with a low mean square error, indicating strong model accuracy. The dataset was split with a test size of 0.25 for validation.

Machine learning outcomes for the substrate height ‘E’ are illustrated in Fig. 11, where the parameter values range from 1.6 to 2.0 μm. The outputs include both actual and predicted data, yielding R² values of 0.49, 0.77, 0.78, 0.89, and 0.87, respectively. The corresponding mean square error is calculated as 1.26.

**Fig. 12:**
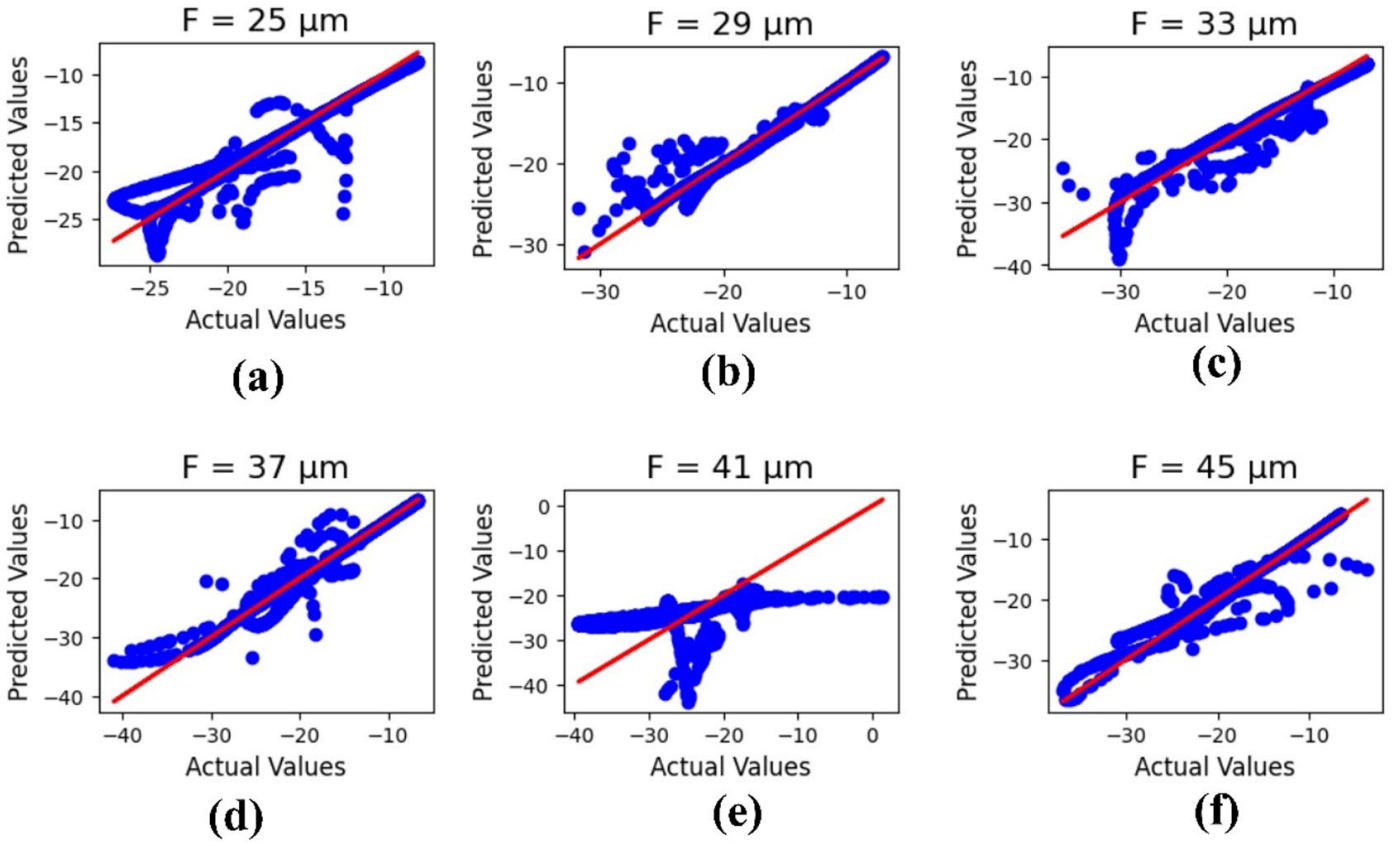
ML-based optimization results for ground plane width variations, shown for parametric values in micrometers: (**a**) 25, (**b**) 26, (**c**) 27, (**d**) 28, (**e**) 29, and (**f**) 30.

Machine learning outcomes for the ground plane width ‘F’ are illustrated in Fig. 12, where the parameter values range from 25 to 45 μm. The outputs include both actual and predicted data, yielding R² values of 0.91, 0.93, 0.92, 0.95, 0.32, and 0.94, respectively. The corresponding mean square error is calculated as 1.72.

**Fig. 13:**
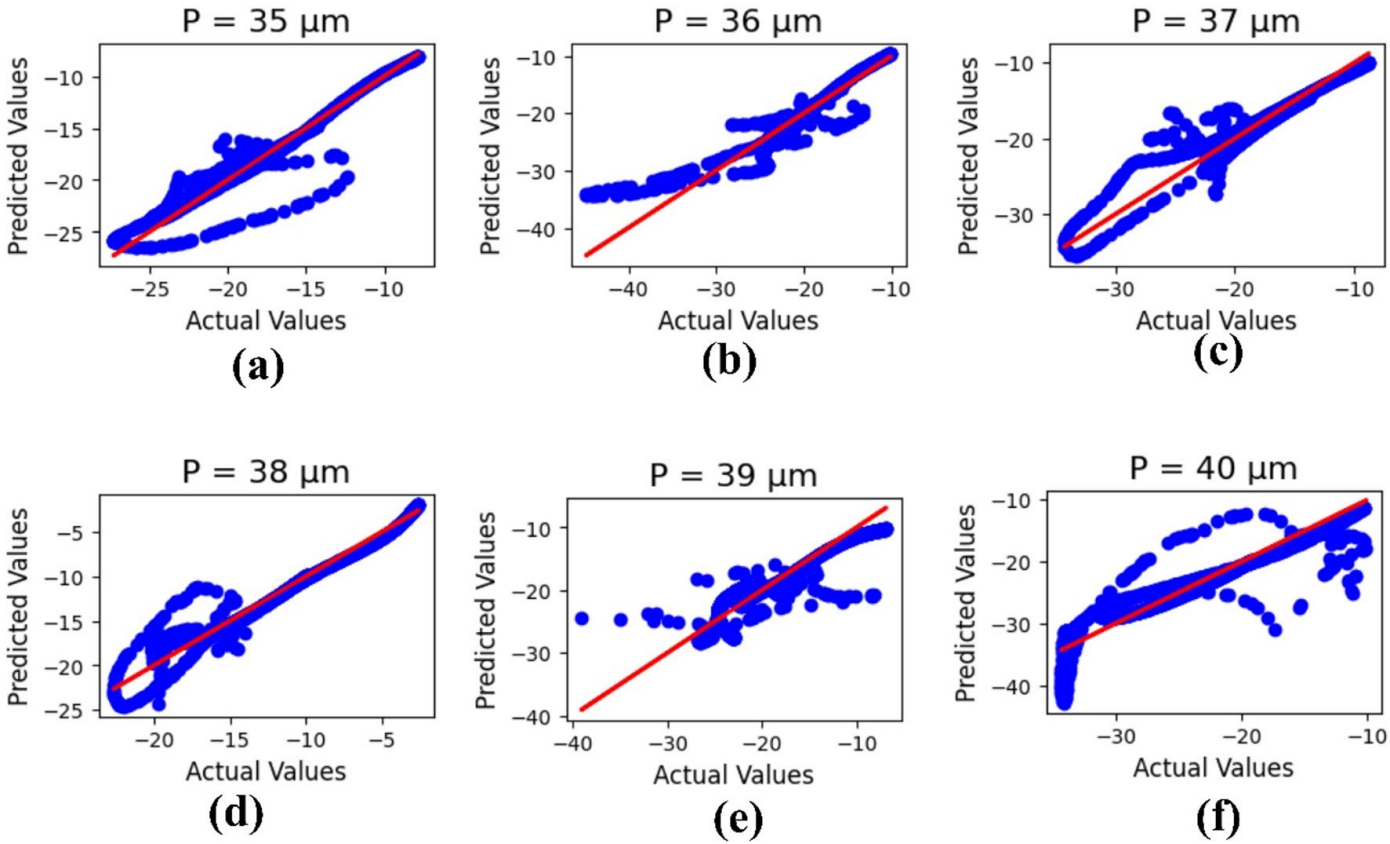
ML-based optimization results for design length variations, shown for parametric values in micrometers: (**a**) 35, (**b**) 36, (**c**) 37, (**d**) 38, (**e**) 39, and (**f**) 40.

Machine learning outcomes for the design length ‘P’ are illustrated in Fig. 13, where the parameter values range from 35 to 40 μm. The outputs include both actual and predicted data, yielding R² values of 0.93, 0.87, 0.88, 0.94, 0.81, and 0.82, respectively. The corresponding mean square error is calculated as 1.53.

**Fig. 14:**
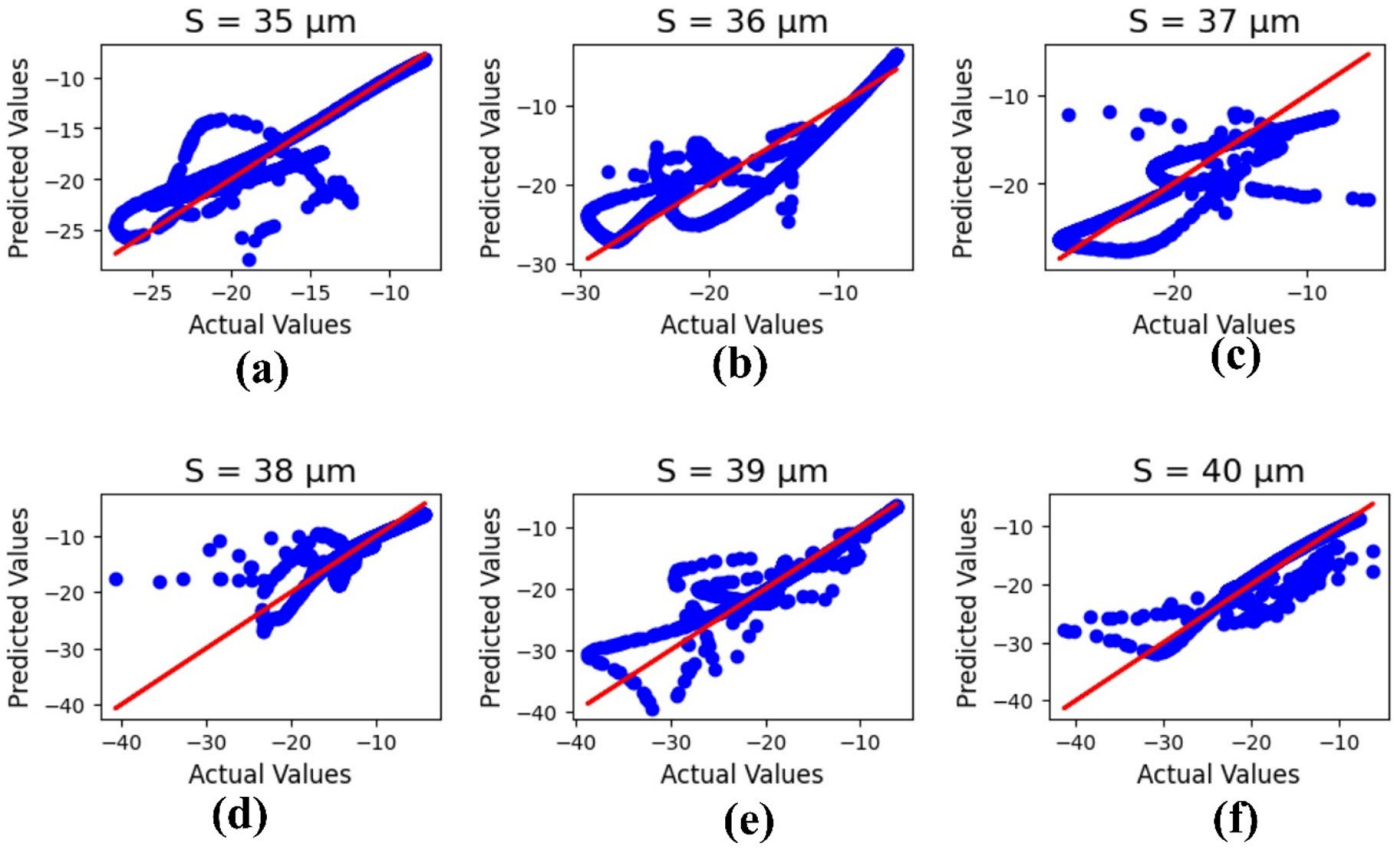
ML-based optimization results for design width variations, shown for parametric values in micrometers: (**a**) 35, (**b**) 36, (**c**) 37, (**d**) 38, (**e**) 39, and (**f**) 40.

Machine learning outcomes for the design width ‘S’ are illustrated in Fig. 14, where the parameter values range from 35 to 40 µm. The outputs include both actual and predicted data, yielding R² values of 0.78, 0.81, 0.74, 0.75, 0.83, and 0.79, respectively. The corresponding mean square error is calculated as 4.59.

## Conclusion

A compact metamaterial-inspired MIMO antenna working in the terahertz range has been designed and analyzed for next-generation high-speed wireless communication systems. The operating range of 10 THz to 70 THz of frequency have been consider in the proposed work. The highest gain of 7.6 dBi and ultra broad-bandwidth of 54 THz is achieved for the proposed antenna. Machine learning optimization is also showing higher optimization efficiency with R^2^ value of 0.95. The proposed antenna demonstrates excellent performance across key MIMO parameters, including low ECC (< 0.005), low TARC ( < − 10 dB), and minimal CCL (< 0.2 bits/s/Hz). The DG maintains a steady value near 10 dB, while the MEG is balanced around − 3 dB, indicating efficient signal reception. These results confirm the antenna’s ability to provide strong isolation, enhanced gain, and wideband operation—attributes that are essential for reliable and efficient THz communication. The integration of metamaterials contributes significantly to bandwidth enhancement and gain improvement, making the proposed antenna a promising candidate for future THz-enabled wireless applications. The suggested MIMO antenna can be expanded further with the inclusion of reconfigurable components to allow dynamic beam steering and frequency agile next generation THz communication systems. The incorporation of enhanced materials and adaptive machine learning systems provides opportunity to have a greater robustness and real time optimization in real deployments.

## Data Availability

The data supporting the findings in this work are available from the corresponding author with reasonable request.

## References

[1] Ali, A. et al. Design process of a compact Tri-Band MIMO antenna with wideband characteristics for sub-6 GHz, Ku-band, and Millimeter-Wave applications. *Ain Shams Eng. J.* **15** (3), 102579. 10.1016/j.asej.2023.102579 (2024).

[2] Jain, R., Thakare, V. V. & Singhal, P. K. Design and Comparative Analysis of THz Antenna through Machine Learning for 6G Connectivity. *IEEE Lat Am. Trans.*10.1109/TLA.2024.10412032 (2024).

[3] Ikram, M., Sultan, K., Lateef, M. F. & Alqadami, A. S. M. A Road towards 6G Communication—A Review of 5G Antennas, Arrays, and Wearable Devices. *Electron. (Switzerland)*. 10.3390/electronics11010169 (2022).

[4] Rasilainen, K., Phan, T. D., Berg, M., Parssinen, A. & Soh, P. J. Hardware Aspects of Sub-THz Antennas and Reconfigurable Intelligent Surfaces for 6G Communications. *IEEE J. Sel. Areas Commun.*10.1109/JSAC.2023.3288250 (2023).

[5] Yadav, R., Gotra, S., Pandey, V. S. & Kumar, S. Graphene based two-port MIMO yagi-uda antenna for THz applications. *Micro Nanostruct.*10.1016/j.micrna.2023.207616 (2023).

[6] Patel, S. K. & Baz, A. O-Shape Fractal Antenna Optimized Design with Broad Bandwidth and High Gain for 6G Mobile Communication Devices. *Fractal Fract.*10.3390/fractalfract8010017 (2024).

[7] Singh, G., Sandha, K. S. & Kansal, A. GA based optimized graphene antenna design for detection of explosives and drugs using THz spectroscopy. *Micro Nanostruct.*10.1016/j.micrna.2023.207566 (2023).

[8] Hussain, M. et al. Metamaterials and Their Application in the Performance Enhancement of Reconfigurable Antennas: A Review. *Micromachines* **14** (2), 349. 10.3390/mi14020349 (2023). 36838049 10.3390/mi14020349 PMC9964562

[9] Hui, W., Guo, Y. & Zhao, X. Polarization-Tunable Microstrip Antenna Based on Double V-Type Metamaterials Cover for Microwave Energy Harvesting. *IEEE Antennas Wirel. Propag. Lett.*10.1109/LAWP.2022.3223404 (2023).

[10] Saleh, A. M., Elmesalawy, M. M., Mahmoud, K. R. & Ibrahim, I. I. Impact of different finite MIMO array geometries on system throughput with considering mutual coupling and edge effect between array elements. *Ain Shams Eng. J.*10.1016/j.asej.2021.03.002 (2021).

[11] Khan, D., Ahmad, A. & Choi, D. Y. Dual-band 5G MIMO antenna with enhanced coupling reduction using metamaterials. *Sci. Rep.* **14** (1), 96. 10.1038/s41598-023-50446-0 (2024). 38168470 10.1038/s41598-023-50446-0 PMC10761735

[12] Hasan, M. M. et al. A Compact Mu-Near-Zero Metamaterial Integrated Wideband High-Gain MIMO Antenna for 5G New Radio Applications. *Mater. (Basel)*. 10.3390/ma16041751 (2023). 10.3390/ma16041751 PMC9961439 36837381

[13] Vasu Babu, K., Das, S., Varshney, G., Sree, G. N. J. & Madhav, B. T. P. A micro-scaled graphene-based tree-shaped wideband printed MIMO antenna for terahertz applications. *J. Comput. Electron.*10.1007/s10825-021-01831-3 (2022).

[14] Babu, K. V. et al. Design and optimization of micro-sized wideband fractal MIMO antenna based on characteristic analysis of graphene for terahertz applications. *Opt. Quantum Electron.*10.1007/s11082-022-03671-2 (2022).

[15] Pandey, A., Singh, A. P. & Kumar, V. Design and optimization of circularly polarized dielectric resonator-based MIMO antenna using machine learning for 5G Sub-6 GHz. *AEU - Int. J. Electron. Commun.*10.1016/j.aeue.2023.154558 (2023).

[16] Li, W. T., Sen Tang, H., Cui, C., Hei, Y. Q. & Shi, X. W. Efficient Online Data-Driven Enhanced-XGBoost Method for Antenna Optimization. *IEEE Trans. Antennas Propag.*10.1109/TAP.2022.3157895 (2022).

[17] Haque, M. A. et al. Broadband high gain performance MIMO antenna array for 5 G mm-wave applications-based gain prediction using machine learning approach. *Alexandria Eng. J.* **104**, 665–679. 10.1016/j.aej.2024.08.025 (2024).

[18] Smith, D. R., Vier, D. C., Koschny, T. & Soukoulis, C. M. Electromagnetic parameter retrieval from inhomogeneous metamaterials. *Phys. Rev. E*. **71** (3), 036617. 10.1103/PhysRevE.71.036617 (2005). 10.1103/PhysRevE.71.036617 15903615

[19] Nicolson, A. M. & Ross, G. F. Measurement of the Intrinsic Properties Of Materials by Time-Domain Techniques. *IEEE Trans. Instrum. Meas.*10.1109/TIM.1970.4313932 (1970).

[20] Al-Bawri, S. S., Abdulkawi, W. M., Sheta, A. A. & Moniruzzaman, M. A High‐Performance 3D Eight‐Port THz‐MIMO Antenna System Verified With Machine Learning for Enhanced Wireless Communication Systems. *Int. J. Commun. Syst.*10.1002/dac.6006 (2025).

[21] Saxena, G., Awasthi, Y. K. & Jain, P. High Isolation and High Gain Super-Wideband (0.33-10 THz) MIMO Antenna for THz Applications. *Optik (Stuttg)*. 10.1016/j.ijleo.2020.165335 (2020).

[22] Younssi, M., Jaoujal, Y., Diallo, E., Moussaoui & Aknin, N. Study of a Microstrip antenna with and without superstrate for terahertz frequency. *Int. J. Innov. Appl. Stud.* (2013).

[23] Kavitha, K. D. R. & Anchitaalagammai, J. V. A graphene based multi-band antenna array for next generation IoT networks in THz spectrum. *Phys. Scr.* **100** (3), 035543. 10.1088/1402-4896/adb4ae (2025).

[24] Zhang, B., Jornet, J. M., Akyildiz, I. F. & Wu, Z. P. Mutual coupling reduction for ultra-dense multi-band plasmonic nano-antenna arrays using graphene-based frequency selective surface. *IEEE Access.*10.1109/ACCESS.2019.2903493 (2019).33224696

[25] Tiwari, R. N. et al. A low-profile sixteen elements four port MIMO antenna array for multiband millimeter wave and conformal electronics. *Millim. Terahertz Waves*10.1007/s10762-025-01093-1 (2025).

[26] Das, S., Mitra, D. & Bhadra Chaudhuri, S. R. Fractal loaded planar Super Wide Band four element MIMO antenna for THz applications. *Nano Commun. Netw.*10.1016/j.nancom.2021.100374 (2021).

[27] Kushwaha, R. K., Karuppanan, P. & Malviya, L. D. Design and analysis of novel microstrip patch antenna on photonic crystal in THz. *Phys. B Condens. Matter*. 10.1016/j.physb.2018.05.045 (2018).

[28] Khaleel, S. A., Hamad, E. K. I., Parchin, N. O. & Saleh, M. B. MTM-Inspired Graphene-Based THz MIMO Antenna Configurations Using Characteristic Mode Analysis for 6G/IoT Applications. *Electronics* **11** (14), 2152. 10.3390/electronics11142152 (2022).

[29] Vettikalladi, H. et al. Sub-THz Antenna for High-Speed Wireless Communication Systems. *Int. J. Antennas Propag.*10.1155/2019/9573647 (2019).

[30] Singhal, S. Elliptical ring terahertz fractal antenna. *Optik (Stuttg)*. 10.1016/j.ijleo.2019.163129 (2019).

[31] Amraoui, Y., Halkhams, I., El Alami, R., Jamil, M. O. & Qjidaa, H. Terahertz dual-band antenna design with improved performances using FSS-based metasurface concept for wireless applications. *Sci. Afr.* **27**, e02566. 10.1016/j.sciaf.2025.e02566 (2025).

[32] Haque, M. A. et al. Performance improvement of THz MIMO antenna with graphene and prediction bandwidth through machine learning analysis for 6G application. *Results Eng.* **24**, 103216. 10.1016/j.rineng.2024.103216 (2024).

[33] Benlakehal, M. E. et al. Design and analysis of MIMO system for THz communication using terahertz patch antenna array based on photonic crystals with graphene. *Opt. Quantum Electron.* **54** (11), 693. 10.1007/s11082-022-04081-0 (2022).

[34] Hossain Nirob, J. et al. Dual-band MIMO antenna for wideband THz communication in future 6G applications, TELKOMNIKA (Telecommunication Comput. *Electron. Control*10.12928/telkomnika.v23i2.26553 (2025).

[35] Singhal, S. Tetradecagonal ring shaped terahertz superwideband MIMO antenna. *Optik (Stuttg)*. 10.1016/j.ijleo.2019.164066 (2020).

[36] Ahmed, M. K. et al. Apr., Graphene-based THz antenna with a wide bandwidth for future 6G short-range communication. *TELKOMNIKA (Telecommunication Comput. Electron. Control*. **23** (2), 306. (2025). 10.12928/telkomnika.v23i2.26562

[37] Tiwari, R. N., Singh, P. & Kumar, P. Graphene based dual band MIMO antenna for THz applications. In *6th International Conference on Communications, Information, Electronic and Energy Systems (CIEES)* 1–4. 10.1109/CIEES66347.2025.11300214 (IEEE, 2025).

[38] Zubair, M. et al. A high-performance sub-THz planar antenna array for THz sensing and imaging applications. *Sci. Rep.* **14** (1), 17030. 10.1038/s41598-024-68010-9 (2024). 39043989 10.1038/s41598-024-68010-9 PMC11266484

